# Preconditioning of Adipose-Derived Mesenchymal Stem-Like Cells with Eugenol Potentiates Their Migration and Proliferation In Vitro and Therapeutic Abilities in Rat Hepatic Fibrosis

**DOI:** 10.3390/molecules25092020

**Published:** 2020-04-26

**Authors:** Moustafa Fathy, Motonori Okabe, Eman M. Othman, Heba M. Saad Eldien, Toshiko Yoshida

**Affiliations:** 1 Department of Regenerative Medicine, Graduate School of Medicine and Pharmaceutical Sciences, University of Toyama, Toyama 930-0194, Japan; moustafa_fathyy@yahoo.com (M.F.); okabe@med.u-toyama.ac.jp (M.O.); 2 Department of Biochemistry, Faculty of Pharmacy, Minia University, Minia 61519, Egypt; eman@toxi.uni-wuerzburg.de; 3 Department of Bioinformatics, Biocenter, University of Würzburg, Am Hubland, 97074 Wuerzburg, Germany; 4 Department of Anatomy, College of Medicine, Jouf University, Jouf, Saudi Arabia; hebasaadeldien2015@yahoo.com

**Keywords:** adipose tissue-derived MSCs, eugenol, migration, self-renewal, hepatic fibrosis, CCl_4_

## Abstract

Mesenchymal stem cells (MSCs) have considerable therapeutic abilities in various disorders, including hepatic fibrosis. They may be affected with different culture conditions. This study investigated, on molecular basics, the effect of pretreatment with eugenol on the characteristics of adipose tissue-derived MSCs (ASCs) in vitro and the implication of eugenol preconditioning on the in vivo therapeutic abilities of ASCs against CCl_4_-induced hepatic fibrosis in rats. The effect of eugenol on ASCs was assessed using viability, scratch migration and sphere formation assays. Expressions of genes and proteins were estimated by immunofluorescence or qRT-PCR. For the in vivo investigations, rats were divided into four groups: the normal control group, fibrotic (CCl_4_) group, CCl_4_+ASCs group and CCl_4_ + eugenol-preconditioned ASCs (CCl_4_+E-ASCs) group. Eugenol affected the viability of ASCs in a concentration- and time-dependent manner. Eugenol improved their self-renewal, proliferation and migration abilities and significantly increased their expression of *c-Met*, reduced expression 1 (*Rex1*), octamer-binding transcription factor 4 (*Oct4*) and *nanog* genes. Furthermore, E-ASCs showed more of a homing ability than ASCs and improved the serum levels of ALT, AST, albumin, total bilirubin and hyaluronic acid more efficient than ASCs in treating CCl_4_-induced hepatic fibrosis, which was confirmed with histopathology. More interestingly, compared to the CCl_4_+ASCs group, CCl_4_+E-ASCs group showed a lower expression of inducible nitric oxide synthase (*iNOS*), monocyte chemoattractant protein-1 (*MCP-1*), cluster of differentiation 163 (*CD163*) and tumor necrosis factor-α (*TNF-α*) genes and higher expression of matrix metalloproteinase (*MMP*)-9 and *MMP-13* genes. This study, for the first time, revealed that eugenol significantly improved the self-renewal, migration and proliferation characteristics of ASCs, in vitro. In addition, we demonstrated that eugenol-preconditioning significantly enhanced the therapeutic abilities of the injected ASCs against CCl_4_-induced hepatic fibrosis.

## 1. Introduction

New era for the treatment of many disorders have been presented by mesenchymal stem cells (MSCs) and natural compounds. MSCs have considerable therapeutic abilities in various disorders, including hepatic fibrosis [1,2,3]. Their easily accessible characteristics and their self-renewal potential make them a promising candidate in regenerative medicine [4,5,6,7]. They have obvious immune-modulatory and migratory capabilities, which are controlled by different factors regulating their maintenance and self-renewal. They are hypo-immunogenic, could manage aroused inflammatory processes, express immunologic suppression factors and do not secrete human leukocyte antigen (HLA) class II [8,9,10,11]. Yet the MSCs homing molecular mechanism is not clearly understood. However, they may be affected with their tissue microenvironment and culture conditions that could affect their characteristics and self-renewal abilities, which in sequence limit their beneficial therapeutic outcomes [12,13,14]. Therefore, careful estimation of the characteristics of MSCs maintained in culture is essential for reserving their potentials. So, new strategies in order to enhance these conditions are necessary to present valuable therapeutic outcomes.

There are exaggerated inflammatory responses during chronic hepatic disorders resulting in hepatic fibrosis, which may lead to cirrhosis, cancer and patients requiring hepatic transplantation, if inappropriately managed [15,16,17]. Since hepatic transplantation is limited, new therapeutic strategies are required for those patients with hepatic fibrosis [18]. Eugenol, a natural compound, which has various concentration-dependent pharmacological activities, regulates many molecular targets like the inhibition of NF-KB activation, down-regulation of prostaglandin synthesis, attenuation of Cox-2 activity, promotion of cell cycle arrest, generation of reactive oxygen species (ROS), diminishing Bcl-2 level and reduction of inflammatory cytokines level [19,20,21]. Effects of many compounds, natural or synthetic, have been examined trying to optimize effective culture conditions that enhance the characteristics of MSCs. It was reported that maintenance, migration and self-renewal characteristics of embryonic stem cells (ESCs) and MSCs were worthily affected by antioxidants, in vitro and in vivo [22]. Furthermore, it was revealed that pretreatment with eugenol improved migration of bone marrow-derived MSCs in vitro [19]. Therefore, it is a promising approach to manipulate MSCs characteristics by adjusting their culture conditions.

In this regard, we previously maintained the stemness characteristics of human Wharton’s jelly mesenchymal cells, established and characterized immortalized mesenchymal and epithelial stem-like cells derived from human amniotic membrane [10,23,24]. Furthermore, we demonstrated that characteristics of adipose tissue-derived mesenchymal stem-like cells (ASCs) maintained in the culture were aberrantly changed [12]. Recently, we reported that the intraperitoneal injection of eugenol enhanced the antifibrotic activity of ASCs through modulation of TGF-beta/Smad signaling pathway [25]. As extension, the present study, for the first time, investigated, on molecular basics, the effect of pretreatment with eugenol on the self-renewal, migration and proliferation characteristics of ASCs in vitro and the implication of eugenol-preconditioning on the in vivo therapeutic abilities of ASCs in the carbon tetrachloride- (CCl_4_-) induced hepatic fibrosis rat model.

## 2. Results

### 2.1. Characterization of Markers Expression in the Isolated ASCs

Characterization of ASCs for the expression of cell surface markers by flow cytometry (FCM) was performed [25]. Furthermore, ASCs were examined by qRT-PCR for the gene expression of MSCs markers (CD90, CD105 and CD73) and hematopoietic markers (CD34 and CD45). Relative to the expression of glyceraldehyde 3-phosphate dehydrogenase (*GAPDH*) mRNA, 65.3% ± 2.9%, 51.7% ± s2.4% and 58.2% ± 3.1% were the expression of CD90, CD105 and CD73 markers, respectively. Low expression of CD34 (3.1% ± 0.9%) and CD45 (9.5% ± 1.1%) was observed.

### 2.2. Viability of ASCs with Eugenol

**Figure 1:**
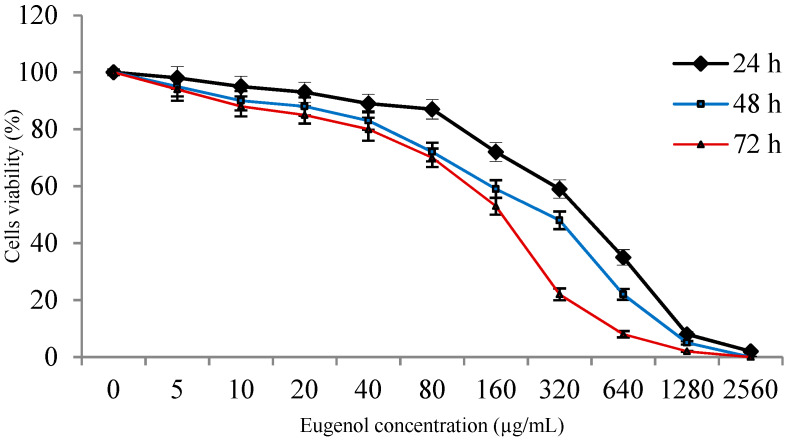
Effect of eugenol on the viability of adipose tissue-derived mesenchymal stem-like cells (ASCs). The viability of ASCs after treatment for 24, 48 and 72 h with different concentrations of eugenol was shown. Viability was expressed as percentage relative to that of eugenol non-treated cells. Data represent mean ± SD. ASCs: adipose tissue-derived mesenchymal stem-like cells.

The viability of ASCs after treatment with different eugenol concentrations for 24, 48 and 72 h was shown in Figure 1. The viability was reduced in a concentration- and time-dependent manner. The results showed that at eugenol concentrations 5, 10 and 20 µg/mL, viability was 98% ± 4%, 95% ± 3.6% and 93% ± 3.5%, respectively, after treatment for 24 h revealing no cytotoxicity at these low concentrations. For the rest of the study, ASCs were pretreated with 10 µg/mL of eugenol for 24 h.

### 2.3. The In Vitro Migration Ability of ASCs is Improved by Eugenol

**Figure 2:**
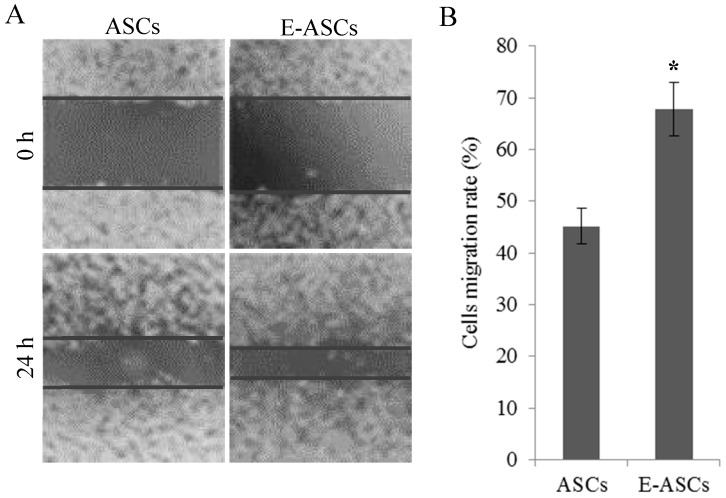
Effect of eugenol on migration ability of ASCs in vitro. (**A**) Representative photos at 0 and 24 h after incubating the cells with normal medium (ASCs) or eugenol-containing medium (E-ASCs). (**B**) Migration ability of the cells. Bars represent mean ± SD. Significant difference is analyzed by a Student’s *t*-test, where: *: *p* < 0.01. ASCs: adipose tissue-derived mesenchymal stem-like cells, E-ASCs: adipose tissue-derived mesenchymal stem-like cells treated with eugenol for 24 h.

To estimate the effect of eugenol on the in vitro migration ability of ASCs, the scratch healing assay was used. Compared to the eugenol-untreated ASCs, E-ASCs significantly (*p* < 0.01) migrated and filled the scratched area after 24 h of eugenol treatment indicating the improvement of ASCs migration ability by eugenol treatment, as shown in Figure 2. 

### 2.4. Effect of Eugenol on Self-Renewal Characteristics of ASCs

**Figure 3:**
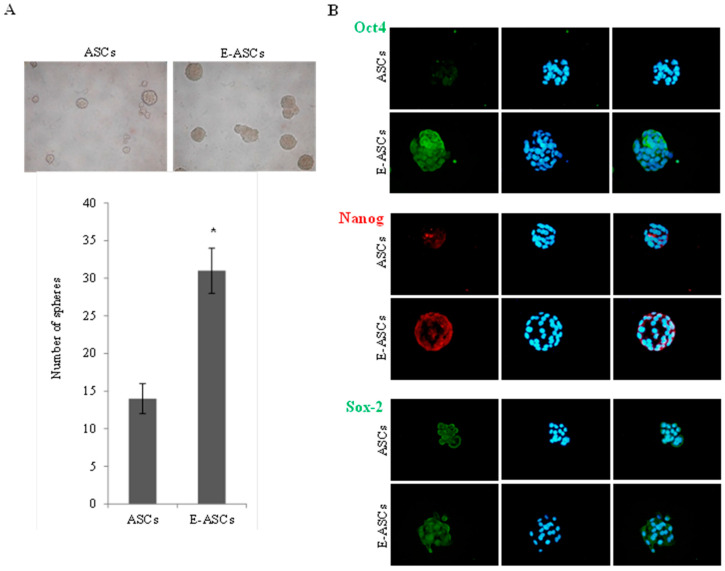
Effect of eugenol on the self-renewal characteristics of ASCs. (**A**) Representative photos for spheres formed after 21 days, magnification: ×40. The ability to form spheres was expressed by the number of the formed spheres. Bars represent mean ± SD. Significant difference is analyzed by a Student’s *t*-test, where: *: *p* < 0.001. (**B**) Immunofluorescence staining of the formed spheres using *Oct4*, *Nanog* and Sox2 antibodies (left lane). Spheres were counterstained with Hoechst (middle lane) then merged (right lane), magnification: ×100. ASCs: adipose tissue-derived mesenchymal stem-like cells, E-ASCs: adipose tissue-derived mesenchymal stem-like cells pretreated with eugenol for 24 h, *Oct4*: octamer-binding transcription factor 4.

Sphere formation assay was used to estimate the effect of eugenol on the sphere formation ability of ASCs. Figure 3A showed that E-ASCs formed 31 ± 3 spheres (with average size 110 ± 7 µm) while ASCs formed 14 ± 2 spheres (with average size 75 ± 5 µm) indicating that the sphere formation ability of eugenol-pretreated cells was significantly (*p* < 0.001) increased compared to the untreated cells. Furthermore, as shown in Figure 3B, spheres formed from E-ASCs expressed octamer-binding transcription factor 4 (*Oct4*) and *Nanog* proteins stronger than those formed from ASCs, but no obvious difference was observed in the expression of the Sox2 protein.

### 2.5. Gene Expression of ASCs Treated with Eugenol

**Figure 4:**
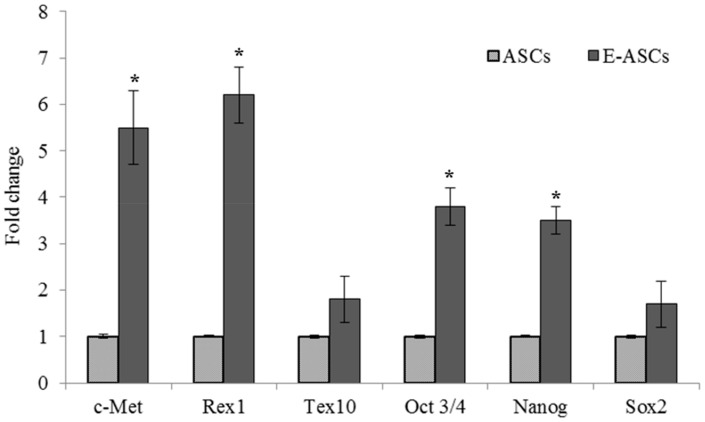
Gene expression of ASCs treated with eugenol. Quantitative RT-PCR was used to evaluate the effect of eugenol on ASCs expression. Data represent fold change relative to ASCs expression after normalization to GAPDH. Bars represent mean ± SD. Significant difference is analyzed by a Student’s *t*-test, where: *: *p* < 0.001. ASCs: adipose tissue-derived mesenchymal stem-like cells, E-ASCs: adipose tissue-derived mesenchymal stem-like cells treated with eugenol, Rex1: reduced expression 1, Tex10: testis-expressed 10, Oct4: octamer-binding transcription factor 4.

Quantitative RT-PCR was used to evaluate the effect of eugenol on ASCs expression for *c-Met,* reduced expression 1 (*Rex1*), testis-expressed 10 (Tex10), *Oct4*, *Nanog* and *Sox2* genes, which are involved in migration, proliferation and self-renewal characteristics. Figure 4 showed that eugenol improved the expression of these genes. Compared to ASCs, the expression of *c-Met*, *Rex1*, *Oct4* and *Nanog* genes in E-ASCs was significantly (*p* < 0.001) increased to become 5.5 ± 0.8, 6.2 ± 0.6, 3.8 ± 0.4 and 3.5 ± 0.3 folds, respectively, while the expression of Tex10 (1.8 ± 0.5) and Sox2 (1.7 ± 0.5) genes was increased but none significantly.

### 2.6. Homing Ability of ASCs is Enhanced by Eugenol

**Figure 5:**
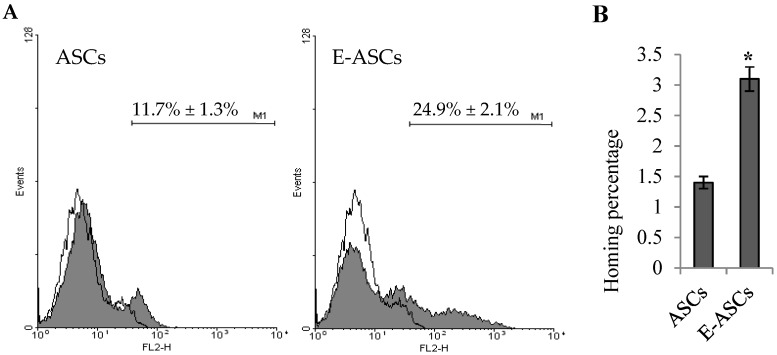
Hepatic homing ability of ASCs is enhanced by eugenol. (**A**) Representative histograms of the flow cytometric analysis for hepatic tissues of rats injected with CM-Dil-labeled ASCs or CM-Dil-labeled E-ASCs. (**B**) The percentage of homing. Bars represent mean ± SD. Significant difference is analyzed by a Student’s *t*-test, where: *: *p* < 0.001. ASCs: adipose tissue-derived mesenchymal stem-like cells, E-ASCs: adipose tissue-derived mesenchymal stem-like cells preconditioned with eugenol.

After labeling with CM-Dil and cellular injection, flow cytometry was used to assess the hepatic homing ability of ASCs or E-ASCs. As shown in Figure 5, hepatic tissues of rats injected with ASCs presented (11.7% ± 1.3%) of CM-Dil labeling while those of rats injected with E-ASCs showed (24.9% ± 2.1%). The hepatic homed cells percentage of E-ASCs (3.1% ± 0.2%) was significantly (*p* < 0.001) increased compared to that of ASCs (1.4% ± 0.1%). So, eugenol-pretreatment enhanced the homing ability of ASCs.

### 2.7. Serum Biochemical Investigations

Serum biochemical investigations, for transaminases, albumin, total bilirubin and hyaluronic acid, were performed to evaluate hepatic function, damage and fibrosis at the end of the experiment after treating the fibrotic rats with ASCs or E-ASCs. As shown in Table 1, CCl_4_ significantly (*p* < 0.001) increased the levels of alanine transaminase (ALT), aspartate transaminase (AST), total bilirubin and hyaluronic acid in the serum of fibrotic rats and reduced albumin level. When compared to the CCl_4_ fibrotic group, treatment with ASCs significantly changed the measured parameters (*p* < 0.05 for ALT and AST, *p* < 0.01 for total bilirubin, hyaluronic acid and albumin), while treatment with E-ASCs showed higher significant changes (*p* < 0.01 for ALT, AST and hyaluronic acid, *p* < 0.001 for total bilirubin and albumin). In all serum levels of the investigated biochemical parameters, there is a significant difference between CCl_4_+ASCs and CCl_4_+E-ASCs groups.

### 2.8. Gene Expression for Rats of Different Groups

**Figure 6:**
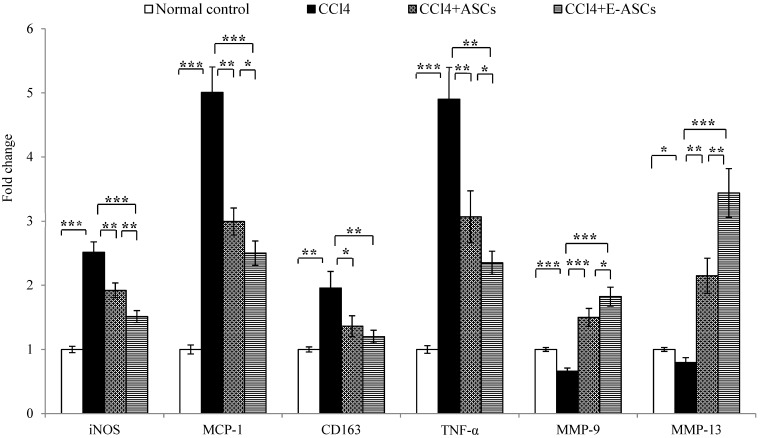
Gene expression in hepatic tissues for rats of different groups. Quantitative RT-PCR was used to evaluate the gene expression in hepatic tissues for rats of different groups. Data represent fold change relative to the normal control group expression after normalization to glyceraldehyde 3-phosphate dehydrogenase (GAPDH). Bars represent mean ± SD. Significant difference between groups is analyzed by a one-way ANOVA test, where: *: *p* < 0.05, **: *p* < 0.01 and ***: *p* < 0.001. ASCs: adipose tissue-derived mesenchymal stem-like cells, E-ASCs: adipose tissue-derived mesenchymal stem-like cells preconditioned with eugenol, CCl4: carbon tetrachloride, iNOS: inducible-nitric oxide synthase, MCP-1: monocyte chemoattractant protein-1, CD163: cluster of differentiation 163, TNF-α: tumor necrosis factor-α, MMP: matrix metallopeptidase.

Gene expression in hepatic tissues of rats was estimated after treating the fibrotic rats with ASCs or E-ASCs by qRT-PCR. In this study the expressions of inducible nitric oxide synthase (*iNOS*), monocyte chemoattractant protein-1 (*MCP-1*), cluster of differentiation 163 (*CD163*), tumor necrosis factor-α (*TNF-α*), matrix metalloproteinase (*MMP*)-9 and *MMP-13* genes were estimated. Figure 6 revealed that in rats of the fibrotic group, compared to the normal control group, CCl_4_ significantly up-regulated the expression of *iNOS* (*p* < 0.001), *MCP-1* (*p* < 0.001), *CD163* (*p* < 0.01) and *TNF-α* (*p* < 0.001) genes and down-regulated *MMP-9* (*p* < 0.001) and *MMP-13* (*p* < 0.05) genes expression. On the other hand, when compared to CCl_4_ group, rats of CCl_4_+ASCs group, treated with ASCs, showed a significant decrease in the expression of genes elevated by CCl_4_ (*p* < 0.01 for *iNOS*, *MCP-1* and *TNF-α*; *p* < 0.05 for CD163) and significant increase in *MMP-9* (*p* < 0.001) and *MMP-13* (*p* < 0.01) genes expression. 

Furthermore, the treatment with E-ASCs showed better improvement in the expression of all investigated genes. More interestingly, in the CCl_4_+E-ASCs group, expression levels were significantly changed compared to those in the CCl_4_+ASCs group (*p* < 0.01 for *iNOS* and *MMP-13*; *p* < 0.05 for *MCP-1*, *TNF*-*α* and *MMP-9*) but there was no significant change in *CD163* gene expression.

### 2.9. Histopathological Examination for Rats of Different Groups

**Figure 7:**
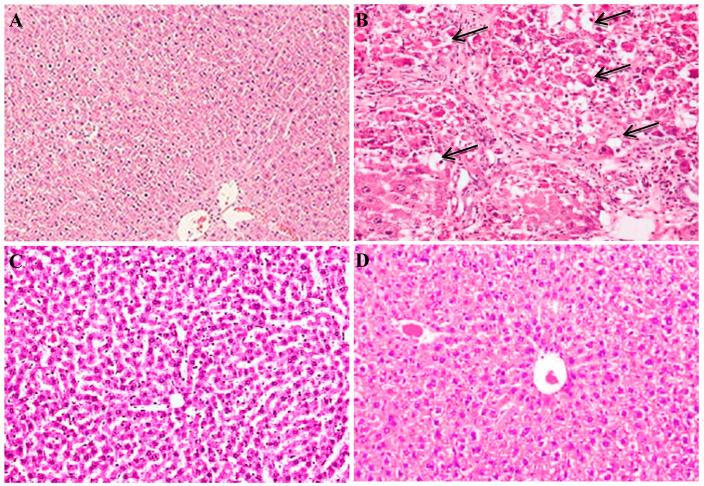
Histological examination of hepatic tissues for rats of different groups. Hepatic tissues were stained with hematoxylin–eosin staining. (**A**) Normal control group, (**B**) fibrotic (CCl4) group, (**C**) CCl4+ASCs group and (**D**) CCl4+E-ASCs group, magnification: ×100. ASCs: adipose tissue-derived mesenchymal stem-like cells, E-ASCs: adipose tissue-derived mesenchymal stem-like cells preconditioned with eugenol, CCl4: carbon tetrachloride.

Hepatic tissues for rats were examined for histopathological changes using hematoxylin–eosin (H&E) staining. As shown in Figure 7, hepatic tissue of fibrotic rats injected with CCl_4_ showed advanced damage with inflammatory infiltration, fatty degeneration and scattered necrotic abnormal cells with distorted tissue architecture. All these changes were markedly reduced after treatment with the cells, especially E-ASCs, showing normal tissue architecture with reduced hepatocyte damage.

## 3. Discussion

Recently, for clinical cell-based therapy, MSCs have got a great attention due to their high therapeutic potential. They were involved in tissue repair, relieved focal cerebral ischemia, enhanced liver function and decreased hepatic fibrosis [4,9,14]. However, their characteristics are influenced by the external culture conditions, so efficient conditions and optimal protocols were investigated trying to maintain or improve the characteristics of MSCs [22]. Regarding bone marrow-derived MSCs, their migration ability was improved by pretreatment with eugenol in an in vitro study [19] while their therapeutic potential against hepatic fibrosis was enhanced by preconditioning with melatonin [26] but nothing was reported regarding MSCs derived from adipose tissue.

Recently, we revealed that the in vitro culture conditions could maintain or affect the stemness and kinetic characteristics of MSCs derived from various organs [12,23]. In addition, we reported the in vivo antifibrotic action of eugenol through the inhibition of iNOS pathway and the modulation of the TGF-beta/Smad pathway [25,27]. Although there are many reports about the pharmacological actions of eugenol [28], there is nothing mentioned about its effect on ASCs behavior in vitro or in vivo. Therefore, this study, for the first time, evaluated not only the in vitro effect of eugenol pretreatment on the characteristics of ASCs, but also the impact of eugenol preconditioning on the therapeutic outcomes of ASCs against CCl_4_-induced hepatic fibrosis in rats. 

In this study, the isolated ASCs were characterized by qRT-PCR. They highly expressed the MSCs markers (CD90, CD105 and CD73) and showed low expression of the hematopoietic markers (CD45 and CD34). The findings of this study showed that eugenol affected the viability of cultured ASCs in a concentration- and time-dependent manner. However until concentration 20 µg/mL, and 24 h treatment, the viability was greater than 93% indicating that no cytotoxicity was observed at these low concentrations while cytotoxicity was observed at higher concentrations at which it may act as a pro-oxidant leading to free radicals production and cellular damage [29,30]. Therefore, we completed the study using eugenol treatment, for 24 h, at low concentration (10 µg/mL) to estimate its effect on ASCs characteristics in vitro and in vivo.

The sphere formation assay and scratch migration assay showed that eugenol improved the self-renewal, proliferation and migration abilities of ASCs. Furthermore, the spheres formed from eugenol pretreated ASCs expressed *Oct4* and *Nanog* proteins stronger than those formed from ASCs as demonstrated by immunofluorescence staining. In addition, qRT-PCR analysis revealed that eugenol significantly increased the expression of *c-Met, Rex1, Oct 4* and *nanog* genes, which are involved in the self-renewal and migration characteristics. *c-Met* expression up-regulation, which was observed after eugenol treatment, may explain the mechanism by which eugenol increased the migratory potential of ASCs. Our study also indicated that the significant up-regulation of *Rex1, Oct 4* and *nanog* genes after eugenol treatment might be responsible for the improvement in self-renewal ability of ASCs. Additionally, the expression of *Tex10* and *Sox2* genes was increased after eugenol treatment but the increase was non-significant.

*Oct4* and *nanog* are transcription factors and components of the pluripotency network. They have essential roles in maintaining the stemness characteristics, in acquiring the self-renewal activity and in differentiation. Previously, we demonstrated the effect of exogenous *Oct4* and *Nanog* on the differentiation ability of human amniotic membrane-derived mesenchymal cells into cardiomyocytes [31,32]. *Rex1*, a novel pluripotency regulator of ESCs, is important for reprogramming and maintaining pluripotency [33,34,35]. It is involved in a transcription factors network in which Oct 4 and *Nanog* up-regulate *Rex1* expression. *Nanog* has been reported to play an important role in sustaining *Rex1* expression [33]. It was shown that proliferation rates were highly proportional with the levels of *Rex1* expression. High expression of *Rex1* in MSCs was reported to increase their multipotency and proliferation ability due to inhibition of p38 MAPK and activation of Wnt/β-catenin [36]. Our study revealed that *Oct4*, *Nanog* and *Rex1* were expressed in ASCs and significantly up-regulated by eugenol treatment suggesting that eugenol may improve the proliferation and self-renewal characteristics of ASCs via the up-regulation of *Rex1* expression, which is mediated by *Oct4* and *Nanog*, leading to Wnt/β-catenin activation.

Furthermore, as extension for our in vitro results, we, in vivo, investigated the effect of eugenol preconditioning on the therapeutic potential of ASCs against hepatic fibrosis induced by CCl_4_ in rats. In this study, the serum levels of ALT, AST, total bilirubin, indicating the hepatocyte damage, and hyaluronic acid, a fibrotic marker [37,38,39], were significantly increased in fibrotic rats while albumin level was decreased indicating hepatocellular damage and fibrosis, which was confirmed with the histopathological examination. Cellular treatment significantly improved all the investigated parameters and the hepatic histopathological changes indicating reduction in hepatic damage and improvement in hepatic function especially in the group treated with E-ASCs in which all the measured parameters were significantly improved compared to the CCl_4_+ASCs group. These results demonstrated the enhancement of the therapeutic potential of eugenol preconditioned ASCs against CCl_4_-induced hepatic fibrosis.

This difference in the improvement between the injected ASCs and E-ASCs may be attributed to the increased homing ability of E-ASCs into the hepatic injured tissue, which is estimated, in this study, by FCM. For cell-based therapy, effective homing of injected cells has been reported as a critical step in the therapy [40,41].

Furthermore, this study demonstrated that effective homing of the injected E-ASCs resulted in attenuation of the expression of genes involved in inflammation, *iNOS, MCP-1, CD163* and *TNF-α*, when assessed by qRT-PCR and up-regulation of the expression of matrix accumulating factors, *MMP-9* and *MMP-13* genes. It was reported before that iNOS and TNF-α expression was highly up-regulated in inflammatory disorders like hepatic fibrosis and attenuated with the treatment [27]. Additionally, MCP-1 was involved in the pathogeneses of different disorders characterized by inflammation and infiltration of monocytes [42,43,44]. Moreover, CD163, which is a marker for monocyte/macrophage lineage, is highly expressed in various inflammatory disorders including hepatic diseases [45,46]. Furthermore, matrix metalloproteinases were involved in cell migration, wound healing, bone development, angiogenesis and embryonic development. During respiratory epithelial and wound healing, MMP-9 was highly up-regulated [47,48]. In addition, MMP-13 was reported as a crucial factor for restructuring the collagen matrix [49]. Additionally, it has been demonstrated that MMP-9 and MMP-13 were involved in the migration of MSCs derived from bone marrow into the hepatic injured tissue [50,51]. Taken these observations together with our results, it was revealed that improvement of the in vivo therapeutic potential of ASCs by eugenol preconditioning may be attributed to increasing their homing ability into the injured hepatic tissue leading to effective attenuation of hepatic inflammation, damage and fibrosis and more ability to reconstruct damaged hepatocytes.

## 4. Materials and Methods

### 4.1. Cells Isolation and Characterization

MSCs were obtained from AT of 6-8 weeks old male rats (180 ± 20 g) as previously described by De Luna-Saldivar et al. [4]. The isolated ASCs were cultured in Dulbecco’s modified eagle’s medium (DMEM, Sigma-Aldrich, St Louis, MO, USA) containing 10% fetal bovine serum (FBS, Biosolutions International, Melbourne, Australia) and 1% penicillin–streptomycin mixture (Nacalai Tesque, Kyoto, Japan) at 37 °C in a 5% CO_2_ humidified incubator. The medium was changed twice a week. Cells at 80%–85% confluence were trypsinized and passaged until the fourth passage. Then the cells were washed and suspended in PBS for use.

Characterization of ASCs for the expression of the cell surface markers, MSC markers (CD90, CD105 and CD73) and hematopoietic markers (CD34 and CD45), by FCM was performed [25]. Furthermore, characteristics of ASCs for their expression to *CD73*, *CD105*, *CD90*, *CD34* and *CD45* genes were confirmed by qRT-PCR as described later.

### 4.2. Cell Viability Assay

To estimate the effect of eugenol on viability of the cells, ASCs were seeded in 96-well plates (5 × 10^3^ cells/well) for 24 h before treatment. Cell counting kit-8 (Dojindo Co., Kumamoto, Japan) was used, according to manufacturer’s instructions, to estimate the viability at 24, 48 and 72 h after treatments with different concentrations (5–1280 µg/mL) of eugenol (99.0%, Sigma Aldrich Company, St Louis, Missouri, USA) using an HTS Multi-Mode Microplate Reader (BioTek Instruments, Winooski, VT). Cell viability was expressed as percentage relative to that of eugenol non-treated cells.

### 4.3. Scratch Migration Assay

To assess the effect of eugenol on the migration and proliferation of ASCs, cells were seeded in 6-well plates (2 × 10^6^ cells/well) with normal medium for 24 h. After forming a confluent monolayer, using a 1-mL tip a central abrade was made in the monolayer. After washing with PBS, a medium containing eugenol (10 µg/mL) or normal medium was added and the cells were incubated for 24 h. Photos were taken using the optical microscope (Leica DMRBE, Germany) at 0 and 24 h. Migration ability of the cells was evaluated as (distance migrated/original scratch width) × 100%.

### 4.4. Sphere Formation Assay and Immunofluorescence Staining

After incubating the cells with medium containing eugenol (10 µg/mL) for 24 h, the eugenol-treated and untreated ASCs were seeded in 24-well ultra-low attachment plates (5 × 10^3^ cells/well) and cultured using normal medium containing insulin (5 μg/mL), hydrocortisone (50 ng/mL), FGF (20 ng/mL), hEGF (20 ng/mL) and heparin (4 μg/mL) for 21 days, then the number of spheres was counted under a microscope.

Furthermore, spheres were taken and fixed in cold acetone before staining with primary antibodies (Santa Cruz Biotechnology, Inc., CA, USA) against *Oct4*, *Nanog* and Sox2 at 4 °C overnight. Then, they were incubated for 1 h with biotin-conjugated multilinked secondary antibody (Dako Cytomation, Glostrup, Denmark) before the incubation with FITC- or PE-conjugated streptavidin. The spheres were counterstained with Hoechst solution and examined by the optical microscope (Leica DMRBE) in the fluorescence mode using DP Controller Leica LMD Software.

### 4.5. Animals and the Experimental Design

Forty male Sprague-Dawley rats (200 ± 20 g, Japan SLC, Hamamatsu, Japan) were used. Animal care and study protocols were preceded according to the guidelines established by The Experimental Animal Center and Research Ethics Committee, University of Toyama, Japan (A2017MED-44). Hepatic fibrosis was induced by intraperitoneal (i.p.) injection of rats twice a week with 1 mL/kg CCl_4_ diluted in olive oil 1:1 (*v/v*) for eight weeks [26]. ASCs were preconditioned with eugenol (10 µg/mL) for 24 h in normal culture medium. Then, the CM-Dil-labeled ASCs (3 × 10^6^ cells/1 mL PBS), either eugenol-preconditioned or non-preconditioned cells, were infused via the tail vein at the beginning of week 5 [52].

Rats were randomly divided into four groups: Group 1, normal control group; Group 2, CCl_4_; Group 3, CCl_4_ + ASCs (CCl_4_+ASCs) and Group 4, CCl_4_ + eugenol-preconditioned ASCs (CCl_4_+E-ASCs). For homing assessment, eight rats were used 48 h after injection of the cells. At the end of the experiment, end of week 8, after fasting for 12 h, the rats were anesthetized and blood samples were collected. Then, rats were sacrificed and liver samples were collected for analysis.

### 4.6. Flow Cytometry for Homing Assessment

Forty eight hours after injection of the cells, liver samples were homogenized by collagenase IV (Invitrogen, USA). After centrifugation, the suspension was subjected to lysis buffer. The red fluorescence CM-Dil was evaluated in the cells suspended in PBS by FCM using the FACSCalibur flow cytometer (Becton Dickinson, Franklin Lakes, NJ, USA). The percentage of homing = [(A × B)/C] × 100% where A: the percent of Dil-labeled cells, B: the number of injected cells and C: the whole organ cellularity [53].

### 4.7. Biochemical Investigations

According to the manufacturer’s instructions, serum ALT, AST, albumin and total bilirubin were estimated using commercially available kits (Abcam KK, Tokyo, Japan) and ELISA kit was used for the determination of the serum level of hyaluronic acid (AMS Biotechnology Ltd., UK).

### 4.8. Gene Expression Analysis using Quantitative Real-Time PCR

Total RNA was isolated from ASCs, E-ASCs or hepatic tissues using Isogen (Nippon Gene, Tokyo, Japan) according to the manufacturer’s protocol. After treatment with deoxyribonuclease I (DNase I, Sigma-Aldrich, Inc.), cDNA was formed using 1 µg total RNA by PrimeScript™ RT reagent kit (Takara Bio, Kusatsu, Japan) with oligo-dT (Applied Biosystems, Foster City, CA, USA). Transcript levels were measured by real-time PCR using the sequence-specific primers, shown in Table 2. Amplification was performed in an ABI PRISM 7900HT Sequence Detector (Applied Biosystems) using the SYBR Green PCR Master Mix (Applied Biosystems) according to the manufacturer’s instructions. GAPDH was used as an internal control gene. Gene expression levels were determined after normalization to GAPDH as a housekeeping gene.

### 4.9. Histological Examination

After formalin fixation, hepatic sections were dehydrated in ascending grades of ethanol and embedded in paraffin. Then, the sections were stained with H&E and observed for histopathological changes using the optical microscope (Leica DMRBE) using the DP Controller software.

### 4.10. Statistical Analysis

Using Statistical Package for the Social Sciences (SPSS version 16.0), data were analyzed by a Student’s *t*-test after a one-way analysis of variance (ANOVA), and expressed as mean ± standard deviation (SD). *p* < 0.05 was considered statistically significant.

## 5. Conclusions

In conclusion, for the first time, this study revealed that eugenol significantly improved the self-renewal, migration and proliferation characteristics of MSCs derived from adipose tissue, in vitro. In addition, our findings demonstrated that eugenol preconditioning significantly enhanced the therapeutic abilities of the injected ASCs against hepatic fibrosis induced by CCl_4_ in rats. Altogether, these results may be helpful for MSCs-based cell therapy applications and provide a new era for researchers trying to improve MSCs therapeutic outcomes.

## Figures and Tables

**Table 1:** Serum levels of the biochemical parameters investigated for the different groups.

Group	ALT (U/L)	AST (U/L)	T. Bilirubin (mg/dL)	Hyaluronic Acid (µg/L)	Albumin (g/dL)
Normal control	46.1 ± 3.5	57 ± 4.1	0.6 ± 0.03	140 ± 11	4.1 ± 0.29
CCl_4_	99 ± 7.2 ^ᶲ^	110.3 ± 8.2 ^ᶲ^	2.3 ± 0.23 ^ᶲ^	310 ± 25 ^ᶲ^	2.5 ± 0.09 ^ᶲ^
CCl_4_+ASCs	81 ± 4.3 ^†^	93.5 ± 5.9 ^†^	1.4 ± 0.1 ^††^	209 ± 10 ^††^	3.3 ± 0.2 ^††^
CCl_4_+E-ASCs	67.2 ± 3.7 ^††,§^	77 ± 4.9 ^††,§^	0.9 ± 0.08 ^†††,§§^	189 ± 9 ^††,§^	3.8 ± 0.19 ^†††,§^

^ᶲ^: *p* < 0.001 vs. normal control group. ^†^, ^††^, ^†††^: *p* < 0.05, *p* < 0.01 and *p* < 0.001, respectively, vs. CCl_4_ group. ^§^, ^§§^: *p* < 0.05 and *p* < 0.01, respectively, vs. CCl_4_+ASCs group. ASCs: adipose tissue-derived mesenchymal stem-like cells, E-ASCs: adipose tissue-derived mesenchymal stem-like cells preconditioned with eugenol, CCl4: carbon tetrachloride, ALT: alanine transaminase, AST: aspartate transaminase, T. Bilirubin: total bilirubin.

**Table 2:** Primers used in qRT-PCR.

Primer	Sequence of the Primer
***CD105***	Forward: 5′-TGGTCTTTTCGAACGAGAATG-3′
	Reverse: 5′-AGCCGGAGGACAATGCTTTTGG-3′.
***CD73***	Forward: 5′-ATAGTCACCTCTGACGATGG-3′
	Reverse: 5′-ATTTCATCTGGGTGTCTGAG-3′.
***CD90***	Forward: 5′-AAGGAGAAACAGGAAACCTC-3′
	Reverse: 5′-ACAGACACAGTCCAACTTCC-3′.
***CD34***	Forward: 5′-AAGATCTTGGGAGCCACCAGAG-3′
	Reverse: 5′-TAGCCCTGGCCTCCACCATTC-3′.
***CD45***	Forward: 5′-TGAACATACGGATTGTGAAA-3′
	Reverse: 5′-TTTGTTCGGACTGTAAGGTT-3′.
***c-Met***	Forward: 5′-GAGGACAAGACCACCGAGGATG-3′
	Reverse: 5′-GGACAACAGCAGCACCAGAATG-3′
***Oct4***	Forward: 5′-CCCAGCGCCGTGAAGTTGGA-3′
	Reverse: 5′-AGAACGCCCAGGGTGAGCCC-3′
***Nanog***	Forward: 5′-CCCTTGCCGTTGGGCTGACA-3′
	Reverse: 5′-AAGGCGGAGGAGAGGCAGTCT-3′
***Sox2***	Forward: 5′-TAGAGCTAGACTCCGGGCGATGA-3′
	Reverse: 5′-TTGCCTTAAACAAGACCACGAAA-3′
***Rex1***	Forward: 5′-ACCCATATCCGCATCCACACC-3′
	Reverse: 5′-GTCTCCACCTTCAGCATTTCTTCC-3′
***Tex10***	Forward: 5′-GCTTGACGGTCGCTCGCTCTC-3′
	Reverse: 5′-TGATGGGTCGGTCGGTCTGTCTC-3′
***iNOS***	Forward: 5′-CACCACCCTCCTTGTTCAAC-3′
	Reverse: 5′-CAATCCACAACTCGCTCCAA-3′
***MCP-1***	Forward: 5′-CTGTCTCAGCCAGATGCAGTTAA-3′
	Reverse: 5′-AGCCGACTCATTGGGATCAT-3′
***CD163***	Forward: 5′-TGTAGTTCATCATCTTCGGTCC-3′
	Reverse: 5′-CACCTACCAAGCGGAGTTGAC-3′
***TNF-α***	Forward: 5′-GGCTCCCTCTCATCAGTTCCA-3′
	Reverse: 5′-CGCTTGGTGGTTTGCTACGA-3′
***MMP-9***	Forward: 5′-CCACTAAAGGTCGCTCGGAT-3′
	Reverse: 5′-GAGTTGCCCCCAGTTACAGT-3′
***MMP-13***	Forward: 5′-TTCTGGTCTTCTGGCACACG-3′
	Reverse: 5′-ATGGGAAACATCAGGGCTCC-3′
***GAPDH***	Forward: 5′-AGACAGCCGCATCTTCTTGT-3′
	Reverse: 5′-TGATGGCAACAATGTCCACT-3′

CD: cluster of differentiation, Oct4: octamer-binding transcription factor 4, Rex1: reduced expression 1, Tex10: testis-expressed 10, iNOS: inducible-nitric oxide synthase, MCP-1: monocyte chemoattractant protein-1, TNF-α: tumor necrosis factor-α, MMP: matrix metallopeptidase, GAPDH: glyceraldehyde 3-phosphate dehydrogenase.
